# Neonatal hereditary spherocytosis: a case report

**DOI:** 10.1186/s13052-025-02049-w

**Published:** 2025-06-17

**Authors:** Carolina Coramusi, Natalia Lucangeli, Sarah Vadalà, Pasquale Parisi, Maria Eleonora Scapillati

**Affiliations:** 1 Faculty of Medicine and Psychology, Sapienza University, Rome, Italy; 2 Neonatal Intensive Care Unit, San Pietro Fatebenefratelli Hospital, Sapienza University, Rome, Italy; 3 Chair of Pediatrics, NESMOS Department, Sant’ Andrea Hospital, Faculty of Medicine & Psychology, Sapienza University, Rome, Italy

## Abstract

**Background:**

Hereditary spherocytosis is a genetic disorder affecting red blood cell membranes, leading to increased destruction and haemolysis. In neonates, it ranges from asymptomatic to severe cases with anaemia, jaundice, and spleen issues. Early diagnosis through clinical, laboratory, and genetic tests is vital for prognosis. This clinical case is presented due to the rarity of neonatal-onset spherocytosis, providing an opportunity for a literature review.

**Case presentation:**

A full-term baby was born via vaginal delivery with a family history of hereditary spherocytosis. The patient was discharged without complications but was later hospitalized for an unrelated issue, during which haemolytic anemia was detected, leading to the beginning of the diagnostic process and subsequent onset of appropriate therapy with a positive outcome.

**Conclusions:**

In cases of neonates with jaundice or anemia, it is crucial to consider neonatal spherocytosis among the differential diagnoses, as early diagnosis allows for appropriate therapy and enables the patient to maintain a normal quality of life.

## Background

Hereditary spherocytosis (HS) is the third most common haemolytic disease after glucose-6-phosphate dehydrogenase deficiency and ABO isoimmunization.

It is observed in all populations, but is particularly common among individuals of northern European, with a prevalence of 0.02 to 0.05%, affecting approximately 1 in 2000 to 1 in 5000 people. HS may account for up to 1% of infants presenting with neonatal jaundice [1]. The primary defect in HS involves the alterations in erythrocyte membrane proteins, primarily ankyrin, leading to impaired membrane deformability and stability; the erythrocytes cannot pass through splenic sinusoids, resulting in high levels of extravascular haemolysis. Among the other proteins involved: band 3 protein, protein 4.2, alpha and beta spectrin [2].

A major challenge in diagnosing HS is its varied clinical presentation. Mild cases may be asymptomatic, while severe cases can cause complications such as splenomegaly, gallstones, and may require blood transfusions. In the absence of a family history, key symptoms to monitor are persistent jaundice, anaemia, and splenomegaly [3]. Anaemia is the most common symptom, in about 50% of cases, ranging from mild to severe, sometimes requiring blood transfusion. Jaundice and/or splenomegaly occur in 10–15% of patients. This variability is partly due to different genes and specific mutations involved, as well as the presence of co-inherited conditions [4].

Early diagnosis is nowadays possible and essential to guarantee timely treatment and ensure a good quality of life for the newborn.

## Case presentation

Baby T was born to a 23-years-old, gravida 0 woman at 38 1\7 weeks’ gestational age. The infant was born at a Level 3 community hospital via vaginal delivery. Apgar’s score was 9–10 at 1–5 min respectively. Birth weight: 2900 gr.

Maternal screens were negative for GBS, HBV, HCV, HIV, CMV, TPHA, and Toxoplasma, and she was Rubella immune. Both maternal and infant blood types were O positive with a negative direct antiglobulin test. The family history revealed autosomal dominant HS, affecting the maternal grandmother and the baby’s mom, who had a haemolytic crisis at six years old and underwent a splenectomy at 16 years old.

The newborn was stable throughout transition and remained in mother-baby care. At 48 h of age he was visibly jaundiced, he had routine total serum bilirubin (TsB) and metabolic screening. The TsB level was 16,5 mg/dl, in the high-risk zone in accordance with the Bhutani nomogram, so phototherapy (PDT) was started and continued for 12 h until he was 60 h old. Baby T was discharged at 72 h of life, with TsB of 11.2 mg/dL, hematocrit (Hct) value of 46% and scheduled for pediatric follow-up.

Unfortunately, at 9 days old, he was hospitalised for rhinitis and dyspnea. During this time, we monitored his hemoglobin (Hb), Hct, and TsB levels. Hb remained at the lower limits, so a follow-up was scheduled for day 22nd.

During the check-up visit the value of Hb was 6.8 g/dL, Hct 18% and TsB 10.7 mg/dl. The patient was admitted to the SubIntensive Neonatal Care ward. Serum lab work including cell blood count (CBC), reticulocyte count, red blood cells (RBC) smear and Parvovirus 19 serology were obtained.

The Hct was 16.4%, Hb was 6 g/dL. CBC showed elevated red cells distribution width (RDW) of 18.7% and a reticulocyte count of 5.29%. mean corpuscular hemoglobin concentration (MCHC) was 36.6 g/dL, mean corpuscular volume (MCV) was 86.3 fL, and the MCHC/MCV ratio was 0.42. RBC smear showed anisopoikilocytosis, hypochromia, macroplatelets, and activated lymphocytes, with no spherocytes. Parvovirus serology was negative.

A transfusion of concentrated RBC was performed. The post-transfusion Hb value was 12.8 g/dL and the Hct 37%.

An abdominal ultrasound revealed a normal-sized spleen (DL: 4.6 cm) with a homogeneous echostructure. Throughout the entire diagnostic and therapeutic course, the neurological findings remained within normal limits for the patient’s age. Muscle tone was well-maintained, there were no episodes of full feeding or other significant neurological clinical events. Additionally, because of the inability of our centre to perform specific diagnostic tests for hemolytic anemias (e.g., EMA binding test, osmotic fragility test, etc.) genetic tests were conducted, with samples collected from the child and parents for genes associated with congenital hemolytic anaemia. Such a decision was made given the uncertain hospitalization duration.

At least, Baby T was discharged at one month of life with Hb 9,9 g/dL, Hct 28%, with instruction to continue therapy with folic acid and follow-up with the paediatric haematology.

There, an EMA binding test was performed, which returned positive, confirming the diagnosis of HS, leaving the genetic test with the exclusive role of diagnostic confirmation.

Subsequently two more blood transfusions were required until the therapy with Erythropoietin (EPO) was started. The child is currently stable and under follow-up at the pediatric hematology clinic.

## Discussion and conclusions

### Methods

A systematic search of electronic databases, with a primary focus on PubMed, was conducted to identify relevant reviews, case reports, and pediatric guidelines published up to 2024 about hereditary spherocytosis and his neonatal onset. Emphasis was placed on literature from the past 10 years.

### Clinical manifestation

Jaundice and neonatal anaemia are conditions frequently managed by neonatologists. Many infants with jaundice undergo PDT. When both conditions are present simultaneously, it is essential to include hemolytic anaemia in the differential diagnosis.

The most common congenital hemolytic anemias in neonates include G6PD deficiency, pyruvate kinase deficiency, and hereditary spherocytosis [4].

In the general diagnosis of HS, the classic triad of anaemia, splenomegaly, and jaundice is typically observed in older children and adults, whereas in newborns it is rare. More than half of neonates with HS may not exhibit anaemia in the first week, and splenomegaly is rarely detected. Jaundice is the most frequent presenting feature. Additionally, neonates often show a sluggish erythropoietic response, resulting in a low reticulocyte count relative to anaemia. Traditional hemolysis markers, such as low haptoglobin levels, may be less reliable indicators of hemolysis in newborns; spherocytes are less commonly seen on blood smears [4].

Spherocytes at the blood smear are identified by the absence of a central zone of pallor, characteristic of normal erythrocytes. These cells have low MCV because of the loss of part of their membrane, with normal Hb content, which leads to an elevated MCHC [5].

In the presence of spherocytes, performing a direct antiglobulin test (DAT) is crucial for differentiating HS from ABO incompatibility. A negative DAT can reasonably exclude ABO incompatibility and suggest HS as the more likely diagnosis.

If present, spherocytes associated with ABO hemolytic disease typically diminish within the first few weeks and usually resolve within 1 to 2 months. In contrast, spherocytes in HS persist beyond this timeframe [1, 5].

### Diagnosis

Diagnosing HS in neonates is challenging, because of the differences in neonatal erythropoiesis, the properties of neonatal erythrocytes, and variations in clinical and laboratory presentations.

One such tool, the HS index, calculated as the mean MCHC/MCV ratio, demonstrated that an index greater than 0.36 is 97% sensitive and over 99% specific for diagnosing dominantly inherited HS in neonates [6–8].

Concurrently, a peripheral blood smear is performed, which documents HS as already described.

The sensitivity and specificity of the previous diagnostic tests in the literature are controversial; therefore, second-line tests are typically performed for diagnostic confirmation [7–9].

Among the second-level tests is the Eosin-5’-maleimide (EMA) binding test (MABT). This test uses EMA, a fluorescent dye, which binds to specific molecules on the RBCs’ membranes, such as band 3. Flow cytometry is then used to measure the fluorescence of the EMA-labelled RBCs, indicating the amount of binding that has occurred [8, 10–12].

The Acidified Glycerol Lysis Time Test measures how quickly red blood cells (RBCs) break down when exposed to a special glycerol solution. This test helps to assess the RBC’s resistance to breaking apart and its fragility. In HS, RBCs have a reduced surface area compared to their volume, leading to rapid breakdown in this test [2, 8, 12].

The Osmotic Fragility Test is used to diagnose HS by evaluating the fragility of RBC. In this test, RBC are exposed to increasingly diluted saline solutions. Normally, cells can tolerate some dilution before breaking apart. In HS, RBC are more fragile because of their altered shape and membrane structure, so they break apart at lower salt concentrations. This increased sensitivity to osmotic pressure helps confirm a diagnosis [8, 11, 12].

Definitive diagnosis is made through genetic testing, looking for pathognomonic genes associated with HS. The most common variants are ANK1, SPTB, SLC4A1, SPTA1, and EPB42, which code for membrane proteins that maintain RBC structure [1, 13, 14].

ANK1, SPTB, and SLC4A1 variants typically follow an autosomal dominant pattern, while SPTA1 and EPB42 defects are often recessive or de novo. Most HS cases in North America and Europe are linked to ANK1 and SPTB gene variants [1, 11, 13, 14].

Traditional diagnostic methods are labour-intensive and costly, including sodium dodecyl sulphate-polyacrylamide gel electrophoresis (SDS-PAGE) and real-time fluorescence quantitative polymerase chain reaction (qPCR) [15]. High-resolution melting curve analysis is fast and can handle many samples at once, but it has limitations in detecting specific base changes and fragment sizes [8, 15].

Next-generation sequencing (NGS) provides a comprehensive approach to gene analysis despite its costs and need for standardization. A focused NGS panel targeting five specific genes can confirm HS diagnosis, enhancing therapeutic management and genetic counselling for affected individuals and families [8, 15].

### Therapy

The life expectancy of these patients depends on the severity of the HS they suffer from and the time of initiation of treatment.

### Phototherapy

The first sign of suspected HS is severe hyperbilirubinemia after birth, which requires intensive PDT and sometimes an exchange transfusion. Therefore, bilirubin levels in newborns suspected of HS should be closely monitored and treated promptly [5].

PDT works by using light to convert bilirubin in the skin into a water-soluble form that can be more easily excreted by the infant’s body. This treatment reduces bilirubin levels and helps prevent the neurological damage that can occur from severe hyperbilirubinemia [4].

PDT involves positioning the newborn 40–50 cm from a light source. Its effectiveness depends on the exposed body surface area, light wavelength (460–490 nm), and TSB levels [4].

Many studies in literature have demonstrated that a significant number of HS cases can be detected among newborns with severe jaundice. Therefore, close monitoring is necessary, especially when there is a slow response to PDT [16].

Breastfeeding should continue if possible; the addition of formula or intravenous hydration is indicated if the baby shows signs of dehydration.

An exchange transfusion might be needed in extreme conditions, when the baby’s jaundice doesn’t improve with PDT. According to the American Academy of Pediatrics, this procedure is recommended if the baby’s total bilirubin level is 25 mg/dL or higher, or if there are signs of serious brain damage from the jaundice (kernicterus) [2].

#### RBC transfusion

In infants with HS, 70–80% need blood transfusions to treat anaemia. The short lifespan of spherocytes can lead to Hb decrease during the first month of life, necessitating one or more RBC transfusions [2, 17].

#### Recombinant erythropoietin

Recombinant erythropoietin (rEPO) is suggested in the literature as an alternative or complementary treatment to blood transfusion. EPO is a glycoprotein hormone that stimulates the production of RBC [1, 2, 4, 16, 17].

The rEPO is used to treat anaemia and the slow production of RBC in newborns. This condition occurs during the first 6 months of life, due to the shift of EPO production from the fetal liver to the neonatal kidneys, and also because of the transition from fetal hemoglobin (HbF) to adult hemoglobin (HbA) [2].

rEPO administration stimulates bone marrow erythropoiesis, as indicated by increased transferrin receptor (TFR) levels, reducing the need for blood transfusions. TFR facilitates iron delivery during cell proliferation and Hb synthesis, making it a reliable biomarker of bone marrow activity [1, 17].

However, due to the high cost and logistical demands of the therapy—twice the cost of a transfusion and requiring injections three times weekly—larger randomized trials are needed to determine best practices [4].

#### Folic acid supplementation

Folate deficiency is common in patients with HS, due to the increased rate of erythropoiesis by hemolytic anaemia. Low folate levels hinder RBC maturation. Therefore, folic acid supplementation is recommended for infants with HS [1, 2].

#### Splenectomy

Splenectomy is the definitive therapeutic intervention for HS, the procedure becomes eligible after the age of five [2].

Splenectomy typically resolves symptoms in almost all patients, by removing the primary site of RBC destruction. It fully corrects anaemia and hyperbilirubinemia, normalizes reticulocyte counts and restores RBC lifespan. In rare cases, where symptoms are not completely resolved, splenectomy still reduces hemolysis [4].

However, the main limitation is the risk of post-splenectomy fulminant sepsis (SFPE), primarily from encapsulated organisms: about 60% from *Streptococcus Pneumoniae*, 25% to *Neisseria Meningitidis* or *Haemophilus Influenzae*. The risk is higher in patients with more severe underlying conditions and younger age. To mitigate these risks, strict adherence to immunisation protocols and prophylactic antibiotic therapy is crucial [2, 4, 12]. 

Splenectomy can be total or partial, this second option preserves some splenic function but may risk regrowth, especially in younger patients. It is sometimes considered in children due to their immature immune systems, which affects antibody production against encapsulated bacteria. However, the literature is sceptical about the surgical approach, and most experts recommend it only for severe cases of HS [1, 4, 12].

## Conclusion

Regarding our patient, the clinical presentation was fully consistent with a suspected diagnosis of HS. On the second day of life, baby T exhibited jaundice that required prolonged PTD. This condition could be considered para-physiological jaundice, and was not associated with anaemia or splenomegaly; consequently, despite the maternal genetic diagnosis, we decided not to perform initial genetic testing for neonatal HS. Additionally, the patient developed severe anaemia by the second week of life, which required RBC transfusion with clinical improvement. Further blood tests, including first and second-level assessments, revealed an elevated MCHC/MCV ratio and a peripheral smear without spherocytes, which is typical for neonates with HS according to the literature. Abdominal ultrasound showed no splenomegaly, as this usually develops later in childhood or adulthood in HS. Genetic testing confirmed the diagnosis of HS with an ANK1 mutation, which is one of the most common, and that is also present in mother and grandmother. The baby responded well to rEPO therapy, did not require additional transfusions so far, and maintained a good growth rate.

In conclusion, HS, although a rare disease, should be considered in the differential diagnosis of common neonatal symptoms such as jaundice and anaemia. It is crucial to initiate first-level laboratory investigations, which can guide the diagnosis (e.g., MCHC/MCV ratio), followed by second and third-level assessments if needed. Additionally, it is essential to investigate the family history for this condition.

## Data Availability

The bibliography for the literature review is entirely available on PubMed.

## Abbreviations

HS: Hereditary spherocytosis
TsB: Total serum bilirubin
PDT: Phototherapy
Hct: Hematocrit
Hb: Hemoglobin
CBC: Cell blood count
RBC: Red blood cells
RDW: Red cells distribution width
MCHC: Mean corpuscular hemoglobin concentration
MCV: Mean corpuscular volume
rEPO: Erythropoietin (EPO)/ Recombinant erythropoietin
DAT: Direct antiglobulin test
MABT: Eosin-5’-maleimide (EMA) binding test
NGS: Next-generation sequencing
